# Transcriptome analysis of sputum cells reveals two distinct molecular phenotypes of “asthma and chronic obstructive pulmonary disease overlap” in the elderly

**DOI:** 10.1186/s40001-022-00861-2

**Published:** 2022-10-28

**Authors:** Suh-Young Lee, Hyun-Seung Lee, Heung-Woo Park

**Affiliations:** 1 Department of Internal Medicine, Seoul National University Hospital, Seoul, Republic of Korea; 2 Department of Internal Medicine, Seoul National University College of Medicine, Seoul, Republic of Korea; 3 Biomedical Research Institute, Seoul National University Hospital, Seoul, Korea; 4 Institute of Allergy and Clinical Immunology, Seoul National University Medical Research Center, Seoul, Republic of Korea

**Keywords:** Asthma, Chronic obstructive pulmonary disease, Asthma-chronic obstructive pulmonary disease overlap syndrome, Sputum, Transcriptome, Cluster analysis

## Abstract

**Background:**

Little is known about the pathogenesis of asthma and chronic obstructive pulmonary disease (COPD) overlap (ACO). This study examined the molecular phenotypes of ACO in the elderly.

**Methods:**

A genome-wide investigation of gene expression in sputum cells from the elderly with asthma, ACO, or COPD was performed using gene set variation analysis (GSVA) with predefined asthma- or COPD-specific gene signatures. We then performed a subsequent cluster analysis using enrichment scores (ESs) to identify molecular clusters in the elderly with ACO. Finally, a second GSVA was conducted with curated gene signatures to gain insight into the pathogenesis of ACO associated with the identified molecular clusters.

**Results:**

Seventy elderly individuals were enrolled (17 with asthma, 41 with ACO, and 12 with COPD). Two distinct molecular clusters of ACO were identified. Clinically, ACO cluster 1 (*N* = 23) was characterized by male and smoker dominance, more obstructive lung function, and higher proportions of both neutrophil and eosinophil in induced sputum compared to ACO cluster 2 (*N* = 18). ACO cluster 1 had molecular features similar to both asthma and COPD, with mitochondria and peroxisome dysfunction as important mechanisms in the pathogenesis of these diseases. The molecular features of ACO cluster 2 differed from those of asthma and COPD, with enhanced innate immune reactions to microorganisms identified as being important in the pathogenesis of this form of ACO.

**Conclusion:**

Recognition of the unique biological pathways associated with the two distinct molecular phenotypes of ACO will deepen our understanding of ACO in the elderly.

**Supplementary Information:**

The online version contains supplementary material available at 10.1186/s40001-022-00861-2.

## Introduction

Asthma and chronic obstructive pulmonary disease (COPD) overlap (ACO) usually refers to a condition characterized by the clinical and inflammatory features of both asthma and COPD. The aging lung undergoes structural changes, immune senescence, and inflammaging, such that the elderly are more susceptible to the development of obstructive airway disease [1]. Accordingly, the prevalence of ACO increases with age [2, 3]. However, whether ACO is a distinct entity remains a matter of debate, due to the lack of knowledge on its pathogenesis.

Sputum is easily obtainable and thus widely used in the study of airway diseases, and sputum cell transcriptomics studies have provided insight into the pathogenesis of asthma and COPD [4, 5]. In addition, recent advances based on machine learning have included comprehensive tools for the analysis of complex transcriptome datasets [6]. Nonetheless, there have been few sputum transcriptomics studies of ACO, especially in the elderly, although the disease is commonly diagnosed in the elderly with respiratory symptoms [7, 8].

In this study, we examined the molecular phenotypes of ACO in the elderly as well as the underlying mechanisms involved in the pathogenesis of this disease. We began by studying genome-wide gene expression in sputum cells from the elderly with asthma, ACO, or COPD, via gene set variation analysis (GSVA) of predefined asthma- or COPD-specific gene signatures. GSVA calculates sample-wise gene set enrichment scores (ESs) as a function of genes inside and outside the gene signature, independent of any class label, and thus allows the underlying pathways in heterogeneous samples to be identified [9]. We then performed a subsequent cluster analysis using ESs obtained from GSVA with asthma- or COPD-specific gene signatures to identify molecular clusters in the elderly with ACO. Finally, the GSVA was repeated using curated gene signatures to gain molecular insights into the pathogenesis of the identified molecular clusters.

## Materials and methods

### Study populations

Participants aged 65 years or older were recruited from the Seoul National University Hospital (Seoul, Korea). Asthma was diagnosed according to the Global Initiative for Asthma guideline (GINA) on the basis of current (past 12 months) episodic respiratory symptoms and demonstrated evidence of airway hyperresponsiveness to methacholine or positive bronchodilator response [10]. The diagnosis of COPD was based on the Global Initiative for Chronic Obstructive Lung Disease (GOLD) guideline [11]. A history of exposure to risk factors, such as tobacco smoking, exposure to environmental tobacco smoke, biomass fuel or occupational exposure to dust, along with the presence of not fully reversible airflow limitation (with or without the presence of symptoms, i.e., the ratio of post-bronchodilator forced expiratory volume in first second [FEV1] and the forced vital capacity [FVC]  < 70% in spirometry) was considered COPD. The 2015 GINA/GOLD documents (the most widely accepted definition) were applied to define ACO [12]. Elderly individuals were diagnosed with ACO based on the presence of a chronic airway disease (medical history of cough, sputum production, wheezing, or repeated lower respiratory tract infections) in combination with features of both asthma and COPD. Smokers were defined as those with a smoking history of  > 10 pack-years. Exclusion criteria were respiratory tract infection, change in maintenance therapy, and exacerbation (short-term oral prednisone burst, unexpected clinic visit, and emergency room visit or hospitalization due to symptom aggravation) within the 4 weeks prior to enrollment. All participants were treated according to the GINA or GOLD guidelines. Sputum induction was done with the patient in a stable state, without the discontinuation of medications, as previously described [13]. Dithiothreitol (0.01 M) was added to sputum samples, vortex mixed, shaken for 20 min at room temperature, filtered through a 52 mm nylon gauze to remove debris and mucus, and then centrifuged at 450 × *g* for 10 min. Cell pellets obtained were resuspended in phosphate buffer saline to a volume equal to the original sputum plus dithiothreitol, After cell viability was determined, a differential cell count was obtained from 300 non-squamous cells [13]. The remainder of this suspension was stored at −80 °C until RNA extraction.

### Gene expression arrays

RNA was extracted from sputum cells using the RNeasy mini kit (Qiagen, Hilden, Germany). RNA quality was measured with the Agilent 2100 bioanalyzer (Biogen, Weston, MA, USA), which performs electrophoretic separations according to molecular weight. Each sample was assigned an RNA integrity number (RIN) based on the extent of RNA degradation. Only samples with RIN values of greater than 8 were placed in aliquots and stored at − 80 °C until microarray, as RIN values of greater than 5 are generally considered adequate for gene expression profiling [14]. Gene expression levels were measured using the GeneChip Human Gene 2.0 ST (Affymetrix, Santa Clara, CA, USA). Probes with poor chromosome annotation and probes in the X or Y chromosome were removed. Then variance-stabilizing transformation and quantile normalization were conducted to reduce the effects of technical noise and to make the distribution of the expression level for each array closer to a normal distribution, respectively.

### Analysis

**Fig. 1:**
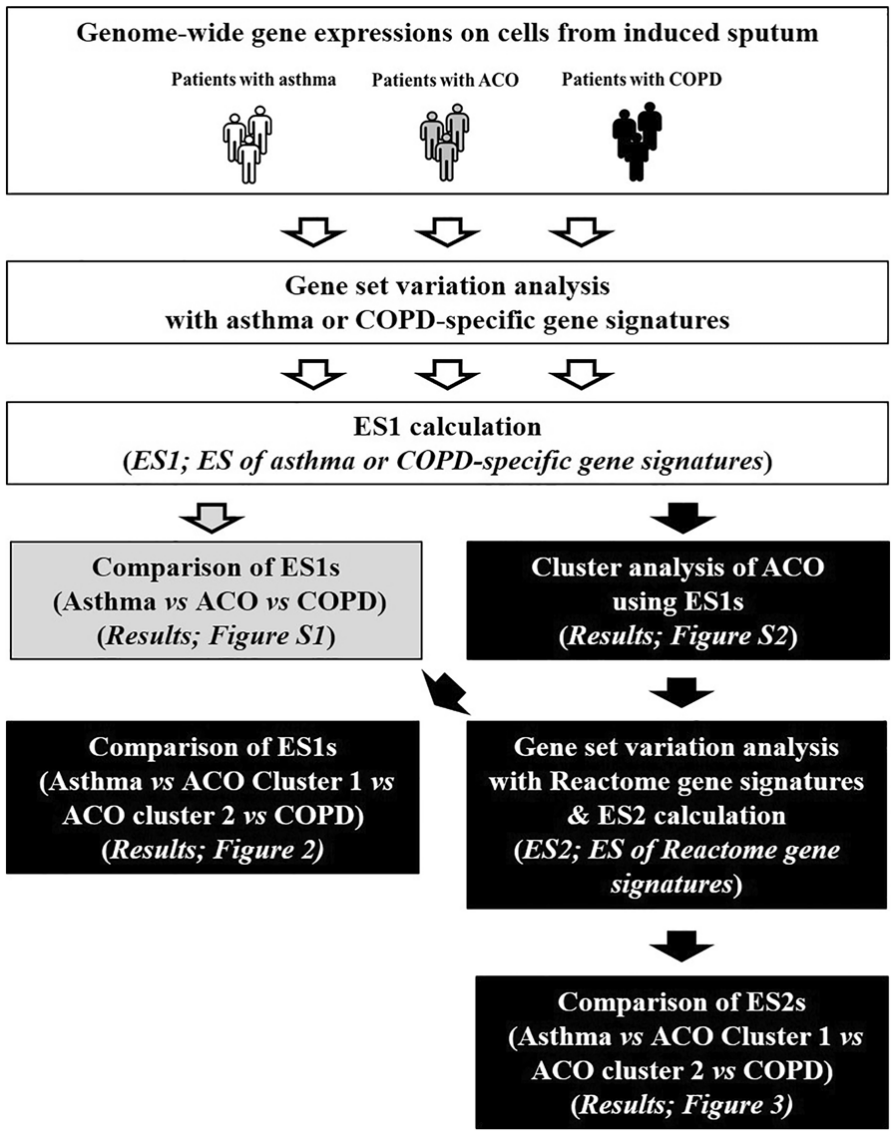
A graphical summary of analysis. *ACO* asthma-COPD overlap, *COPD* chronic obstructive pulmonary disease, *ES* enrichment score

First, the molecular features of ACO were compared to those of asthma or COPD. This was done by defining six gene signatures based on previous reports: the asthma-6 gene signature (six genes obtained from a sputum transcriptome that can discriminate inflammatory phenotypes in asthma [15]); the Th2 gene signature (three genes obtained from an airway epithelium transcriptome that can identify the Th2-high molecular phenotype of asthma [16]); asthma-up and asthma-down gene signatures (67 differentially expressed genes in the sputum of asthmatics compared to healthy controls [64 upregulated and 3 downregulated] [17]); and COPD-up and COPD-down gene signatures (98 differentially expressed genes in the bronchial brushings of patients with COPD compared to healthy controls [54 upregulated and 44 downregulated] [18]). Gene lists of these gene signatures are presented in Additional file 1: Table S1. Then GSVA was performed to evaluate differences in the enrichment of these six gene signatures across whole-sputum gene expression samples. An ES ranging from −1 to + 1 was calculated for each gene signature in patients with asthma, ACO, or COPD. In the next step, a k-means cluster analysis was performed to identify molecular subgroups with ACO, using the ESs obtained from the GSVA based on the six gene signatures. The final number of clusters was determined using a consensus-based method. Finally, to obtain insight into the pathogenesis of ACO associated with the identified molecular clusters, a GSVA was again performed but with the curated gene signatures from the Reactome database. Then the mean ES of each gene signature was compared across all groups (asthma, ACO clusters, and COPD). Examples of biological pathways in the Reactome database include classical intermediary metabolism, signaling, transcriptional regulation, apoptosis, and disease [19]. To minimize the possibility of false positives, only gene signatures with a *P* < 0.05 and a difference in the mean ES (dES) between two groups  > 0.2 were considered significant. All analyses were performed with R version 4.0.3 (www.r-project.org; R Foundation for Statistical Computing, Vienna, Austria). A graphical summary of analysis is presented in Fig. 1.

## Results

**Fig. 2:**
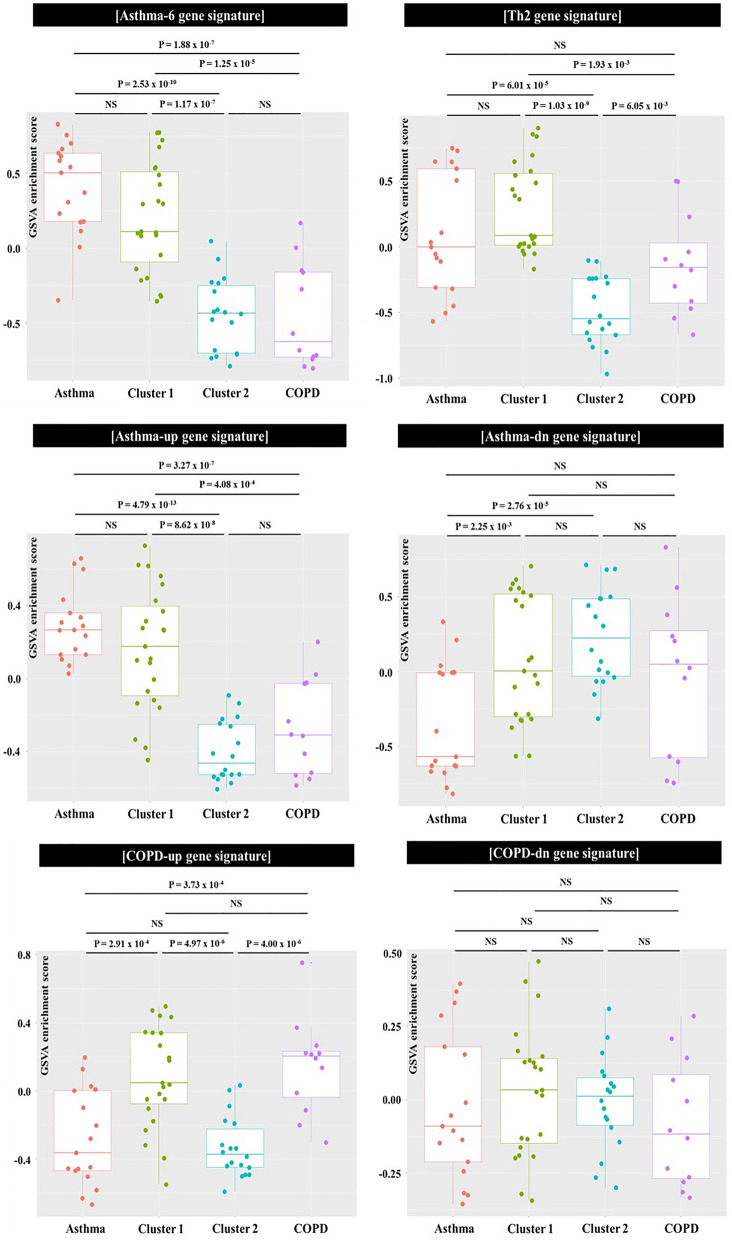
Results of gene set variation analysis using asthma- and COPD- specific gene signatures. The GSVA enrichment scores were calculated across 70 whole-sputum gene expression profiles. ACO clusters 1 and 2 were identified from *k*-means clustering using the enrichments scores obtained from the GSVA with asthma- and COPD-specific gene signatures in the elderly with ACO. Dots represent the individual enrichment scores, and box and whisker plots show the median and interquartile range. Gene lists of asthma- and COPD-specific gene signatures are provided in the online supplement. *GSVA* gene set variation analysis, *ACO* asthma-COPD overlap, *COPD* chronic obstructive pulmonary disease, *NS* not significant

Table 1 summarizes the characteristics of the 70 elderly individuals enrolled in this study (17 with asthma, 41 with ACO, and 12 with COPD). As expected, the clinical features of patients with ACO were between those of patients with asthma and those of patients with COPD. The results of the GSVA are presented in Fig. 2and Additional file 1: Figure S1. Consistent with the clinical characteristics, the molecular features of patients with ACO were also between those of the other groups. Cluster analysis revealed two distinct molecular clusters in the ACO group, with 23 individuals in cluster 1 and 18 in cluster 2 (Additional file 1: Figure S2).

**Table 1:** Characteristics of the elderly individuals enrolled in this study

	Asthma	ACO	COPD	*P* value		
	*N* = 17	*N* = 41	*N* = 12	Asthma vs. ACO	Asthma vs. COPD	ACO vs. COPD
Demographics						
Age, year	73.23 ± 3.54	73.51 ± 5.61	72.58 ± 6.18	NS	NS	NS
Male, N	9 (52.94)	16 (39.02)	8 (66.67)	NS	NS	NS
Height, cm	164.61 ± 6.76	157.44 ± 8.61	160.35 ± 8.81	0.0034	NS	NS
BMI, m^2^/kg	23.45 ± 3.01	25.45 ± 3.78	24.06 ± 3.37	0.015	NS	NS
Smoker, *N*	5 (29.41)	25 (60.97)	12 (100.00)	NS	NA	NA
Medications						
Low dose^*^ ICS, *N*	1 (5.88)	0 (0)	0 (0)	NA	NA	NA
Medium dose^*^ ICS, *N*	13 (76.47)	28 (68.29)	3 (25.00)	NS	0.0074	0.0042
High dose^*^ ICS, *N*	3 (17.65)	13 (31.71)	2 (16.67)	NS	NS	NS
Inhaled LAMA, *N*	0 (0)	35 (85.37)	12 (100.00)	NA	NA	NA
Lung function						
FVC, mL	2771.67 ± 908.34	2489.26 ± 778.25	3378.82 ± 674.35	NS	0.0012	NS
FVCp, %	95 ± 22.67	94.65 ± 18.73	101.82 ± 21.43	NS	0.048	0.037
FEV1, mL	1883.33 ± 557.07	1521.95 ± 484.60	1792.94 ± 384.60	NS	0.032	0.045
FEV1p, %	91.5 ± 23.45	82.31 ± 25.74	75.52 ± 19.32	0.0062	2.7 × 10^−4^	0.014
FEV1/FVC ratio, %	69.25 ± 10.11	62.73 ± 13.97	54.88 ± 12.18	0.0017	1.4 × 10^−4^	0.0098
Inflammatory markers						
Sputum neutrophil, %	33.19 ± 10.90	35.53 ± 13.70	43.76 ± 13.79	NS	0.0036	0.042
Sputum eosinophil, %	6.02 ± 6.70	3.86 ± 6.37	0.97 ± 1.11	0.0027	5.7 × 10^−5^	0.016
Blood eosinophil, %	4.53 ± 2.82	2.60 ± 2.07	3.01 ± 2.31	0.039	NS	NS

Data are presented as the mean ± standard deviation (SD) except sex, smoking status, and medication (number [%])

*ACO* asthma-COPD overlap, *COPD* chronic obstructive pulmonary disease, *N* number, *BMI* body mass index, *ICS* inhaled corticosteroid, *LAMA* long acting muscarinic antagonist, *FVC* forced vital capacity, *FVCp* FVC predicted value, *FEV1* forced expiratory volume in one second, *FEV1p* FEV1 predicted value, *NA* not applicable, *NS* not significant

^*^Based on the Global Initiative for Asthma guideline[10]

In ACO cluster 1, the ESs of the asthma-6, asthma-up, and Th2, and signatures showed no differences compared to the respective ESs in the asthma (Fig. 1). Similarly, the ESs of the COPD-up gene signature in ACO cluster 1 showed no differences compared to the those in the COPD group (Fig. 2). However, in ACO cluster 2, these values were significantly lower than the respective ESs in the asthma and COPD groups (Fig. 2). These results suggest that the molecular features of ACO cluster 1 were similar to those of asthma and COPD. The clinical features of the elderly in ACO cluster 1 and 2 are presented in Table 2. ACO cluster 1 was characterized by male and smoker dominance, more obstructive lung function, and higher proportions of neutrophils and eosinophils in induced sputum.

**Table 2:** Characteristics of ACO clusters 1 and 2 in the elderly

	Cluster 1	Cluster 2	*P* value
	*N* = 23	*N* = 18	
Demographics			
Age, year	73.56 ± 5.42	73.44 ± 6.01	NS
Male, *N*	11 (47.82)	5 (27.78)	NS
Height, cm	157.30 ± 9.04	157.63 + 0 ± 8.28	NS
BMI, m^2^/kg	26.27 ± 4.21	25.73 ± 3.25	NS
Smoker, *N*	19 (82.61)	6 (33.33)	0.0038
Medications			
Medium dose^*^ ICS, *N*	15 (65.22)	13 (72.22)	NS
High dose^*^ ICS, *N*	8 (34.78)	5 (27.78)	NS
Inhaled LAMA	20 (86.96)	15 (83.33)	NS
Lung function			
FVC, mL	2567.82 ± 880.87	2388.89 ± 633.99	NS
FVCp, %	97.52 ± 21.43	91.00 ± 14.61	NS
FEV1, mL	1471.30 ± 384.60	1586.66 ± 460.74	NS
FEV1p, %	79.13 ± 19.32	86.38 ± 24.93	NS
FEV1/FVC ratio, %	59.21 ± 14.75	67.22 ± 11.82	NS (0.068)
Inflammatory markers			
Sputum neutrophil, %	39.40 ± 14.03	30.59 ± 11.87	NS (0.091)
Sputum eosinophil, %	5.34 ± 7.87	1.96 ± 2.89	0.039
Blood eosinophil, %	2.93 ± 2.41	2.18 ± 1.52	NS

Data are presented as the mean ± SD except sex, smoking status, and medications (number [%])

*ACO* asthma-COPD overlap, *COPD* chronic obstructive pulmonary disease, *N* number, *BMI* body mass index, *ICS* inhaled corticosteroid, *LAMA* long acting muscarinic antagonist, *FVC* forced vital capacity, *FVCp* FVC predicted value, *FEV1* forced expiratory volume in one second, *FEV1p* FEV1 predicted value, *NS* not significant

^*^Based on the Global Initiative for Asthma guideline[10]

**Fig. 3:**
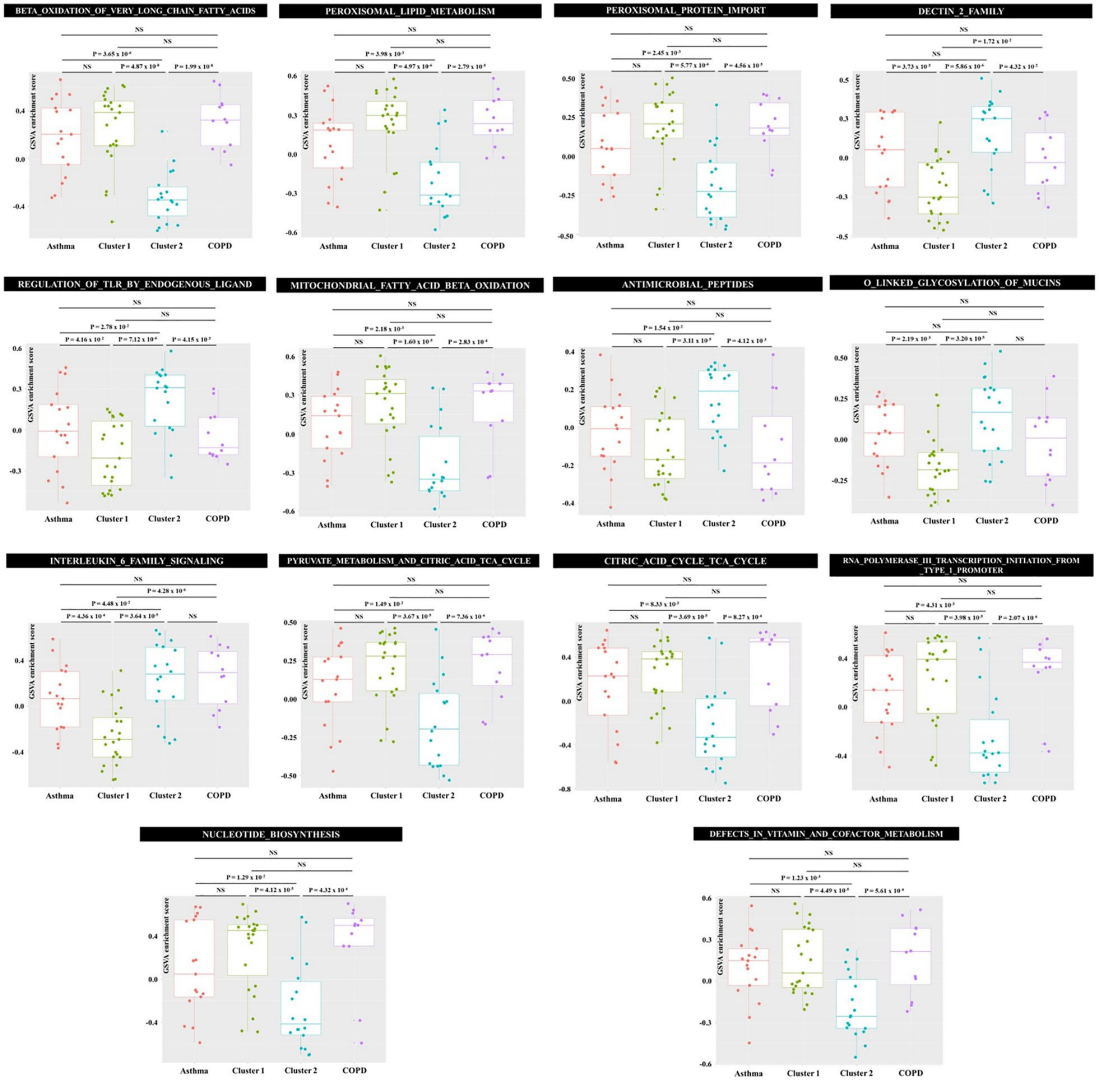
Results of gene set variation analysis using gene signatures from the Reactome database. Dots represent the individual enrichment scores, and box and whisker plots show the median and interquartile range. Gene signatures that were significantly different in their enrichment between ACO cluster 1 and cluster 2 are shown

Next, a GSVA with 1101 gene signatures from the Reactome database was performed to identify biological pathways enriched in each ACO cluster. The minimum and maximum size of the resulting gene sets were 10 and 100, respectively. After Bonferroni correction for multiple tests, the ESs of 14 gene signatures differed significantly between ACO clusters 1 and 2 (*P* < 4.54 × 10^−5^ [= 0.05/1101] and dES > 0.2) (Fig. 3). The enrichment of gene signatures related to mitochondria function and peroxisome function was significantly higher in ACO cluster 1 than in ACO cluster 2 (Fig. 3). However, the ESs of these gene signatures in ACO cluster 1 did not significantly differ from those of asthma or COPD. The pathogenesis of ACO described by ACO cluster 1 may be mechanistically similar to that of asthma or COPD and mainly due to mitochondrial and peroxisome dysfunction. Several gene signatures showed significantly higher enrichment in ACO cluster 2 than in ACO cluster 1, asthma, and COPD. These biological pathways have important roles in innate immunity against various infectious or non-infectious stimuli [20–23]. These results suggest that innate immunity against environmental stimuli, including infection, contributes to the development of ACO defined by cluster 2, by a mechanism independent of that underlying the pathogenesis of asthma or COPD.

Finally, we searched for the clinical relevance of biological pathways identified by evaluating correlations between the ESs of 14 gene signatures and clinical variables (lung function-related variables, proportions of eosinophils and neutrophils in sputum, and the proportion of eosinophils in white blood cells). In ACO cluster 2, a significantly negative association of the Interleukin_6_family_signaling pathway with FVCp (*P* = 0.017), FEV1 (*P* = 0.028), FEV1p (*P* = 0.0013), and the FEV1/FVC ratio (*P* = 0.021) and a significantly positive association of this pathway with the sputum neutrophil levels (*P* = 0.030) were determined (Additional file 1: Figure S3).

## Discussion

The results of a GSVA using asthma- and COPD-specific gene signatures revealed that, consistent with its clinical characteristics, the molecular features of ACO, determined by sputum gene expression, are between those of asthma and COPD. Two distinct molecular clusters of ACO in the elderly were identified using the ESs obtained from the GSVA with asthma- and COPD-specific gene signatures. Clinically, ACO cluster 1 was characterized by male and smoker dominance, more obstructive lung function, and higher proportions of both neutrophils and eosinophils in induced sputum compared to ACO cluster 2. In a subsequent GSVA with curated gene signatures from the Reactome database, 14 gene signatures that differed significantly in their enrichments between ACO cluster 1 and ACO cluster 2 were identified. The ESs of gene signatures related to mitochondria and peroxisome functions were significantly higher in ACO cluster 1 than in ACO cluster 2 but did not differ between ACO cluster 1 and asthma or COPD. However, the ESs of gene signatures related to innate immunity against various stimuli, including microorganisms, were significantly higher in ACO cluster 2 than in ACO cluster 1, asthma, or COPD.

The aging lung makes the elderly more susceptible to the development of obstructive airway diseases [1]. However, while ACO is clinically diagnosed, there is still debate whether it is a distinct entity. We therefore investigated the molecular features of ACO in the elderly using sputum transcriptomics. A recent large-scale genome-wide association study showed that ACO has a spectrum of shared genetic influences, some predominantly influencing asthma and others predominantly influencing fixed airflow obstruction [24]. In addition, changes in gene expression in the airway can co-occur in asthma and COPD, which explains the “asthma-like” features in patients with COPD [25]. Taken together, these findings suggest that, in ACO of the type identified in ACO cluster 1, the genetic mechanisms of the disease are the same as those of asthma or COPD. Indeed, we showed that the enrichment levels of asthma- and COPD-specific gene signatures were similar in ACO cluster 1, asthma, and COPD, indicative of a common genetic pathogenesis, including the involvement of genes related to mitochondria and peroxisome functions. For example, the beta-oxidation of fatty acids in mitochondria plays a crucial role in asthmatic bronchial smooth muscle remodeling and in cigarette smoke-induced COPD [26, 27], and the mitochondrial tricarboxylic acid cycle has been implicated in airway inflammation in asthma and in the pathogenesis of COPD [28, 29]. In addition, peroxisome proliferator-activated receptors, involved in the regulation of inflammatory reactions and lipid metabolism, participate in chronic inflammation in both asthma and COPD [30, 31]. Although mitochondria and peroxisome dysfunctions are not the only mechanisms involved in the pathogenesis of asthma and COPD, they may converge to result in ACO. A previous study of patients with ACO reported mitochondrial dysfunction, characterized by an increase in the mitochondrial DNA/nuclear DNA ratio, although the ratio was slightly closer to that of COPD than that of asthma [32].

The molecular features of ACO cluster 2 were distinct from those of asthma and COPD. The lung is consistently exposed to noxious stimuli, including microorganisms. Although there is a controversy regarding which comes first, infections combine with inflammatory and pathological changes in the development of asthma and COPD [33] and antimicrobial peptides from the airway epithelium have been associated with the pathologic features of both [34, 35]. ACO cluster 2 was characterized by the enrichment of dendritic cell-associated C-type lectine-2 (Dectin-2) family and the TLR regulation by endogenous ligands pathway. Dectin-2 is expressed on myeloid and non-myeloid cells and acts as a non-TLR innate immune receptor [36]. Together, these findings suggest that enhanced innate immune reactions to microorganisms contribute to the development of ACO as described by ACO cluster 2. While clinically significant pulmonary infection was not recognized in the elderly of ACO cluster 2, the human lung is not a sterile organ and culture-independent molecular techniques have demonstrated the presence of microorganisms in the airway [37]. Ours is the first report to show that enhanced innate immune reactions to microorganisms are related to ACO in the elderly. Subsequent studies focusing on microbiome differences between ACO cluster 1 and cluster 2 in the elderly are needed.

We also found significant negative associations between the ESs of the Interleukin_6_family_signaling gene signature in ACO cluster 2 and lung function variables. The IL-6 family of cytokines consists of 10 members, including IL-6, leukemia inhibitory factor, and oncostatin M, all of which share the signal transducer gp130 in their receptor complexes and have overlapping but also distinct biologic activities, such as the hepatic acute phase reaction, B-cell stimulation, the regulation of T-cells, and metabolic regulation [38]. A recent study implicated IL-6 family cytokines in the pathogenesis of both asthma and COPD [39]. The shared relevance of IL-6 cytokines suggest that IL-6 *trans*-signaling plays an important role in these diseases [40, 41]. The ADAM17/IL-6 *trans*-signaling axis in COPD is involved in alveolar cell apoptosis [42]. Oncostatin M is a regulator of the extracellular matrix in many tissues and is therefore likely to play a role in airway remodeling in asthma [43]. Enhanced IL-6 family cytokine signaling may therefore contribute to reduced lung functions in the elderly with ACO and is a potential target of treatment to prevent lung function decline.

There were limitations to this study that should be mentioned. Firstly, the number of participants was small and our findings could not be confirmed in an independent population, both of which limit the generalizability of our results. Secondly, ongoing medications might have affected gene expression profiles in sputum, as participants in this study did not stop their prescribed medications when their sputum was induced. In addition, most participants showed relatively normal lung functions and were treated by a medium-dose inhaled corticosteroid. Our results need to be confirmed again in severe patients with asthma, ACO or COPD. Finally, this study was performed in the elderly, and aging itself results in changes in immune function and lung structure [1]. Thus, whether our observations can be extrapolated to other age groups remains to be determine.

## Conclusions

In this study, we examined the molecular phenotypes of ACO in the elderly using sputum gene expression profiles and identified two distinct clusters. ACO cluster 1 had molecular features similar to those of asthma and COPD, with mitochondria and peroxisome dysfunction as important pathogenetic mechanisms. The molecular features of ACO cluster 2 differed from those of asthma and COPD and included enhanced innate immune reactions to microorganisms as important contributors to disease pathogenesis.

## Supplementary Information

**Additional file 1: Figure S1. **Results of gene set variation analysis using asthma- and COPD-specific gene signatures. **Figure S2.** Cluster plot. **Figure S3.** Correlation plots between the enrichment scores of 14 gene signatures and the clinical variables in ACO clusters 1 and 2. **Table S1.** Gene lists of asthma- and COPD-specific gene signatures.

## Data Availability

The datasets used and/or analyzed during the current study are available from the corresponding author on reasonable request.
