# Supplementary material for: Transcriptome analysis of sputum cells reveals two distinct molecular phenotypes of “asthma and chronic obstructive pulmonary disease overlap” in the elderly
Source: Eur J Med Res. 2022 Oct 28;27:215. doi: 10.1186/s40001-022-00861-2 (PMC9617312; doi:10.1186/s40001-022-00861-2)

**Online supplementary materials**

**Transcriptome analysis of sputum cells reveals two distinct**

**molecular phenotypes of ‘asthma and chronic obstructive pulmonary disease overlap’ in the elderly**

Suh-Young Lee, MD, PhD,^1,2^ Hyun-Seung Lee, PhD,^3^ Heung-Woo Park, MD, PhD,^1,2,4^

^1^Department of Internal Medicine, Seoul National University Hospital, Seoul, Republic of Korea

^2^Department of Internal Medicine, Seoul National University College of Medicine, Seoul, Republic of Korea

^3^Biomedical Research Institute, Seoul National University Hospital, Seoul, Korea

^4^Institute of Allergy and Clinical Immunology, Seoul National University Medical Research Center, Seoul, Republic of Korea

**Figure legends**

**Figure S1. Results of gene set variation analysis using asthma- and COPD- specific gene signatures**

The GSVA enrichment scores were calculated across 70 whole-sputum gene expression profiles. Dots represent the individual enrichment score, and box and whisker plots the median and interquartile range. Gene lists of asthma- and COPD-specific gene signatures are provided in the online supplement. GSVA; gene set variation analysis, ACO; asthma-COPD overlap, COPD; chronic obstructive pulmonary disease, NS; not significant

**Figure S2. Cluster plot**

*k*-means clustering using enrichment scores obtained from the gene set variation analysis using asthma- and COPD-specific gene signatures in the elderly with ACO. These two components explain 75.59% of the point variability.

**Figure S3. Correlation plots between the enrichment scores of 14 gene signatures and the clinical variables in ACO cluster 1 and 2**

A. ACO cluster 1

B. ACO cluster 2

The significant (P < 0.05) correlations determined in a Spearman correlation analysis are presented. Positive correlations are shown in blue and negative correlations are in red. The areas of the circles are proportional to the absolute value of the corresponding correlation coefficient. FVC; forced vital capacity, FVCp; FVC predicted value, FEV1; forced expiratory volume in one second, FEV1p; FEV1 predicted value, Ratio; FEV1/PVC, Neup; neutrophil proportion in induced sputum, Eosp; eosinophil proportion in induced sputum, BECp; eosinophil proportion in white blood cell

**Table S1.** **Gene lists of asthma- and COPD- specific gene signatures**

|  |  |
| --- | --- |
| **Gene Signature** | **Gene** |
| Asthma-up | *CLC, TFF3, FFAR3, BTNL8, VSTM1, CCL17, OLIG1, IL18R1, LGALS12, IL1R2, CD24, DEFA3, GGT5, BPIFB1, TPST1, IL1RL1, CACNG6, VEGFA, CEBPE, ZBTB16, P2RY14, DACH1, PRR7, MXD1, B3GNT8, TNFSF14, TKTL1, PSCA, HIST1H2AD, RAB44, IL5RA, RAMP1, FAM65B, GPR56, SLPI, NFE2, PIGR, MMP25, MSMB, CCR3, WFDC2, SLC25A37, FAM159A, SEMA7A, IL3RA, HIST2H2AA4, THBS1, HIST2H2AB, IL1R1, ANGPTL4, RGL4, CHST15, AGR2, CXCR1, ARAP3, SERPINB3, ADAM8, ZNF395, ASPRV1, TPSAB1, CD69, ASB2, CLEC4G, TIAM2, ICAM3, TMEM71, CSGALNACT1* |
| Asthma-dn | *IFI44L, HP, HPR* |
| Asthma-6 | *CLC, CPA3, DNASE1L3, IL1B, ALPL, CXCR2* |
| Th2 | *CLCA1, SERPINB2, POSTN* |
| COPD-up | *PTGS2, C8orf4, IL1R2, SLCO1B3, GSDMC, GABRP TNC, TMPRSS11D, DSC3, SERPINB13, UGT1A10, CYP2C18, UPK1B, GCNT3, FGFBP1, ARNTL2, CD109, ABCA12, TCN1, FUT3, SRPX2, CEACAM5, PLA2G4A, DPYSL3, GAD1, TPRXL, SLC26A2, DKFZP564O0823, CATSPERB, IRAK3, BCL2L15, ATB10B, LOC400986, C15orf48, MUC4, INDO, MUC13, TRIM31, MIA, IGF2BP3, GLYATL2, SLC5A8, MTNR1A, LCN2, SAA4, SAA2, SAA1, CFB, SLC26A4, CD200R1, NR4A1, FOS, DUSP5, HBB* |
| COPD-dn | *SCGB3A2, CRISP3, GRP, GLDN, IRS4, SERPINB7, LOC653198, CLDN22, PTGFR, ACAA2, GPR116, PEG10, SGCE, MGP, C6, C4orf31, LMO3, NPR3, WIF1, LPR2, GABRG1, VGLL3, EPB41L2, TLL1, TNS3, SLIT2, FAM38B, FHOD3, HSD17B13, KIAA1622, CLDN8, PECI, LTF, AVPR1A, DNAJC5B, SEMA3E, CXorf57, KCNMB2, C6of201, PRUNE2, LRRC63, CORO2B, ZNF382, CCDC81* |

**Figure S1.**


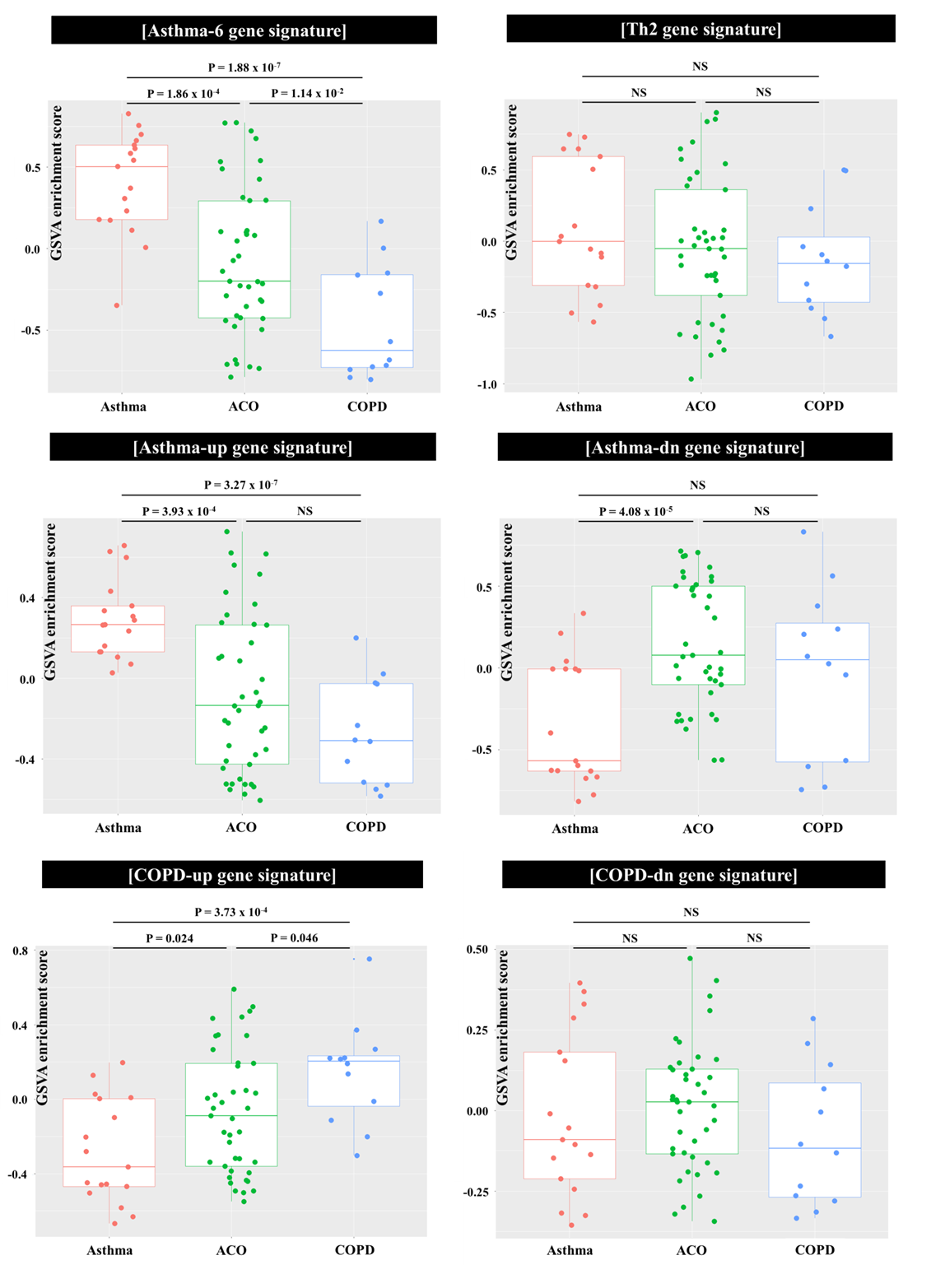


**Figure S2.**


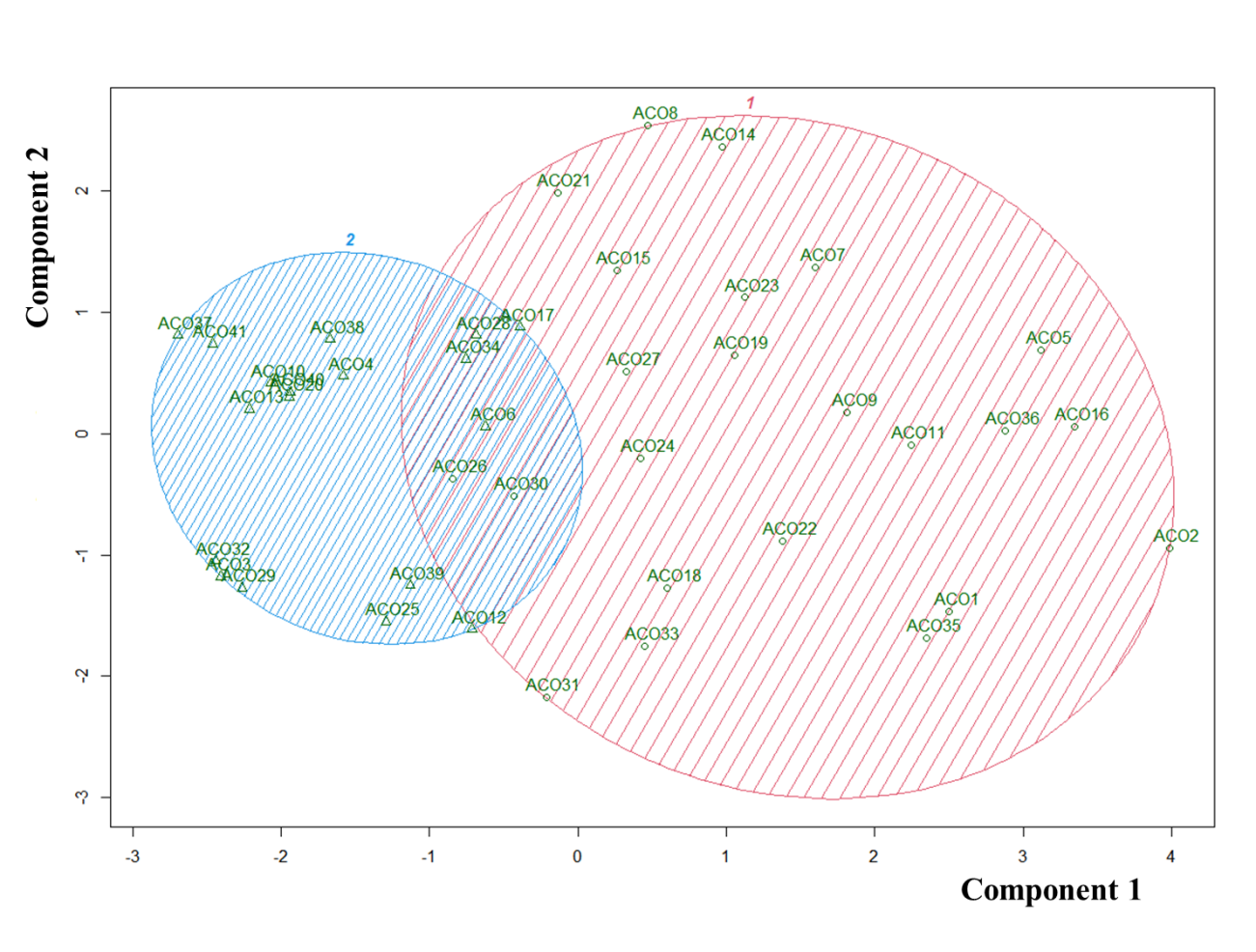


**Figure S3.**

A. B.


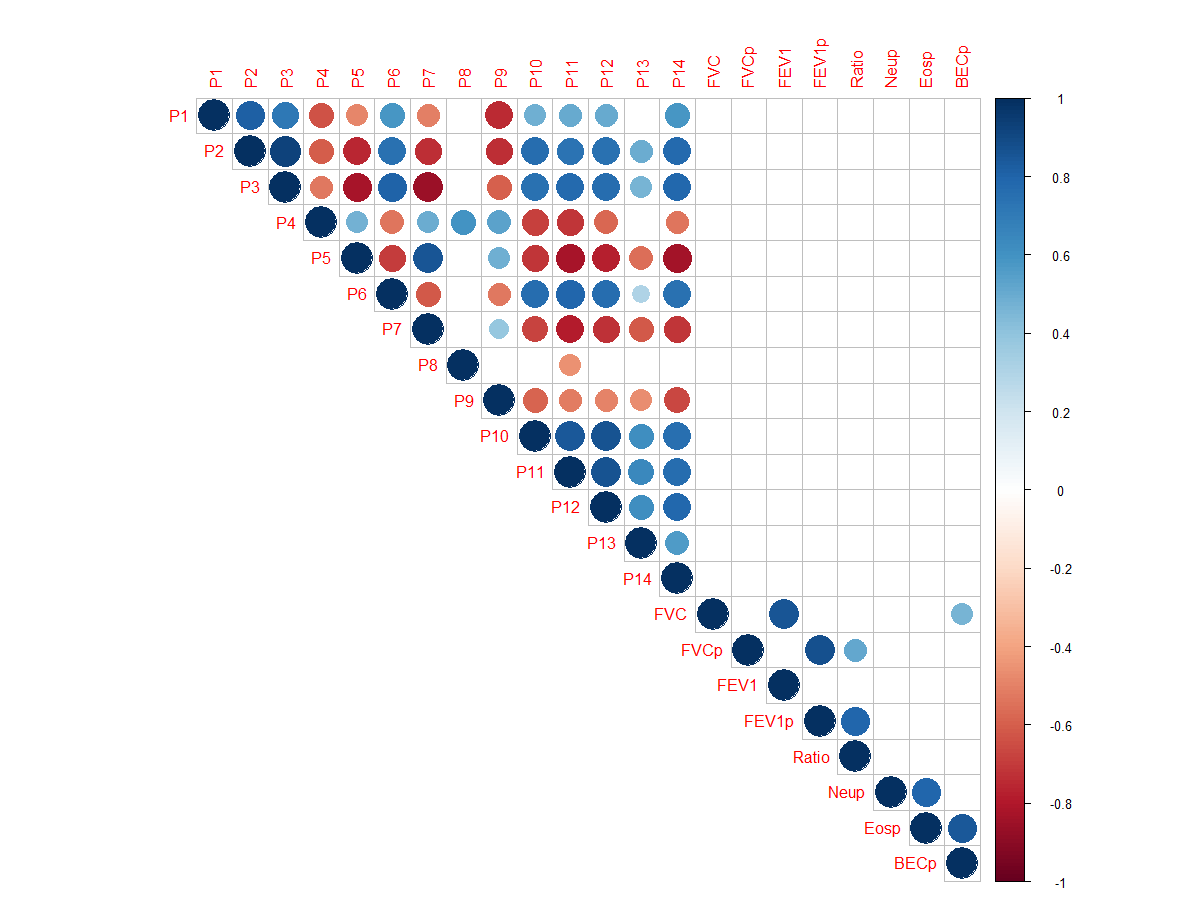

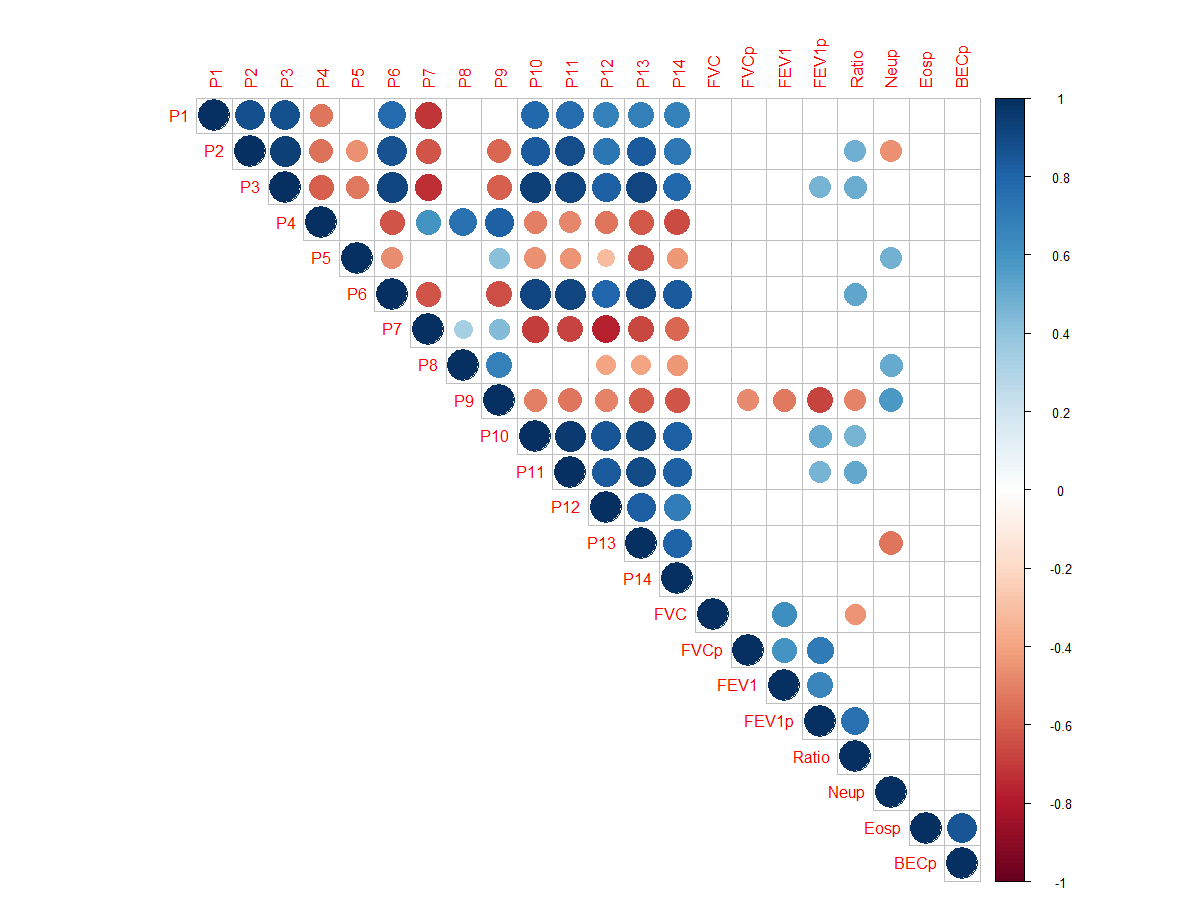

Supplement: Supplementary file 1 — Additional file 1: Figure S1. Results of gene set variation analysis using asthma- and COPD-specific gene signatures. Figure S2. Cluster plot. Figure S3. Correlation plots between the enrichment scores of 14 gene signatures and the clinical variables in ACO clusters 1 and 2. Table S1. Gene lists of asthma- and COPD-specific gene signatures. [file 40001_2022_861_MOESM1_ESM.docx]
